# The expressed needs of people with Chronic Fatigue Syndrome/Myalgic Encephalomyelitis: A systematic review

**DOI:** 10.1186/1471-2458-9-458

**Published:** 2009-12-11

**Authors:** Maria de Lourdes Drachler, Jose Carlos de Carvalho Leite, Lee Hooper, Chia Swee Hong, Derek Pheby, Luis Nacul, Eliana Lacerda, Peter Campion, Anne Killett, Maggie McArthur, Fiona Poland

**Affiliations:** 1 School of Allied Health Professions, University of East Anglia, Norwich, NR4 7TJ, UK; 2 School of Medicine, Health Policy and Practice, University of East Anglia, Norwich, NR4 7TJ, UK; 3 Plaishetts House, Hadspen, Castle Carey, BA7 7LR, UK; 4 London School of Hygiene and Tropical Medicine, Keppel Street, London, WC1E 7HT, UK; 5 Hull and East Yorkshire Medical Research and Teaching Centre, Castle Hill Hospital, Castle Road, Cottingham, HU16 5JQ, UK

## Abstract

**Background:**

We aimed to review systematically the needs for support in managing illness and maintaining social inclusion expressed by people with chronic fatigue syndrome/myalgic encephalomyelitis (CFS/ME)

**Methods:**

We carried out a systematic review of primary research and personal ('own') stories expressing the needs of people with CFS/ME. Structured searches were carried out on Medline, AMED, CINAHL, EMBASE, ASSIA, CENTRAL, and other health, social and legal databases from inception to November 2007. Study inclusion, data extraction and risk of bias were assessed independently in duplicate. Expressed needs were tabulated and a conceptual framework developed through an iterative process.

**Results:**

Thirty two quantitative and qualitative studies, including the views of over 2500 people with CFS/ME with mainly moderate or severe illness severity, met the inclusion criteria. The following major support needs emerged: 1) The need to make sense of symptoms and gain diagnosis, 2) for respect and empathy from service providers, 3) for positive attitudes and support from family and friends, 4) for information on CFS/ME, 5) to adjust views and priorities, 6) to develop strategies to manage impairments and activity limitations, and 7) to develop strategies to maintain/regain social participation.

**Conclusions:**

Although the studies were heterogeneous, there was consistent evidence that substantial support is needed to rebuild lives. Gaining support depends - most importantly - on the ability of providers of health and social care, colleagues, friends and relatives, and those providing educational and leisure services, to understand and respond to those needs.

## Background

Chronic Fatigue Syndrome/Myalgic Encephalomyelitis (CFS/ME) is an idiopathic, long term, multi-faceted, potentially disabling and life-disrupting illness. Its prevalence is at least 0.2-0.4% in the UK[1] There is no specific diagnostic test, and treatment is based on symptom management, aiming to sustain or extend capacity.

Expressed needs are those identified by people with CFS/ME from their experience of living with this condition (as opposed to normative needs, identified by others)[2] Although in-depth understanding of the perspective of service users is fundamental for evidence based policy and practice, insufficient information exists on the expressed needs of people with CFS/ME [1]. Little has been done to draw together the existing data on these expressed needs to support them in managing their illness and maintaining social participation.

We present a systematic review of both first hand accounts of the expressed needs of people with CFS/ME (reported in their narratives of own stories) and researcher-mediated accounts of those needs reported in primary research. The objective was to identify, appraise, select and synthesise what is known about the expressed needs of people with CFS/ME.

## Methods

### Search strategy

No protocol for this review has been published. Searches were carried out on Medline, AMED, CINAHL, EMBASE, ASSIA, The Cochrane Library Clinical Trials Register, Family and Child Law and Employment Law Direct, Health and Psychosocial Instruments, PsycINFO, Health Management Information Consortium, SIGLE, Social Work Abstracts, and the Social Science Index from inception to November 2007. The search was based on ' [text and indexing terms for chronic fatigue] and [text and indexing terms for needs, education, mobility, activities of daily living, legislation, rehabilitation, health services, dysfunction or social behaviour]'. The full Medline search, on which other searches were also based, is shown in Appendix A.

### Criteria for inclusion of studies

Studies were included which had been published in English in peer reviewed journals and reported the needs of people with CFS/ME for support in managing their illness and maintaining social inclusion (excluding specific healthcare interventions such as medication or exercise). Methodologies included:

a) Stories reported by people with CFS/ME themselves (published 'own stories'); or

b) Primary research where researchers set out to assess needs of people with CFS/ME; or

c) Primary research not specifically designed to examine needs (e.g., designed to assess impact or experiences of living with CFS/ME), but where expressed needs of people with CFS/ME were described.

The definitions of CFS/ME were, as in the reviewed papers, based both on professional and patient use of this diagnosis. To be included the needs discussed had to be expressed by people with CFS/ME (in the 'own stories'), in interpretation of their discussions with researchers, or in their responses to questionnaires (in primary research). Studies which additionally included people with other illnesses, people not complaining of illness, family members of people with CFS/ME, their carers or health professionals were also considered, provided needs expressed by people with CFS/ME were reported separately. Where studies were published by the same author(s) and where studies included similar numbers of participants the studies were examined carefully to assess whether they constituted multiple reports from a single study. Where this was the case all of the publications were pooled to provide information on the single study (studies were the unit of inclusion, rather than publications).

### Data collection

An in/out form was used to assess studies for inclusion in the review. Titles and abstracts, and subsequent full text papers, were assessed independently in duplicate and disagreements resolved through discussion with the review team.

A data extraction form was designed to include bibliographic details, study participants, study design and expressed needs (with a quotation from the paper and any relevant interpretation by the reviewer). Original study results were extracted by two reviewers independently. Differences between reviewers' results were resolved by discussion with the review team.

### Quality assessment

The risks of bias of the reviewed papers in accurately reporting needs expressed by people with CFS/ME were assessed independently by two reviewers and, where they disagreed, by a third researcher. Two aspects of the risk of bias were assessed, each aspect being coded as high, medium or low:

***a) Scope for participants to express their needs***: the likelihood that the data collection methods allowed the expression of needs by participants: *high*, if own story, participative action research or naturalistic observation as these prioritise free participant expression; *medium*, if in-depth interview as these support participant expression, *low*, if standardised questionnaire only as these provide limited opportunities for participant expression.

***b) Scope for the needs expressed by the participants to be identified by the reviewer*s**: the likelihood that the data analysis and reporting would convey needs expressed by participants:*high*, if the paper presents participants' narratives or the study results present interpretations of narratives accompanied by quotes from participants; *medium *if interpretation was without quotes; and *low *if statistical analysis only.

### Data syntheses

The reviewers (MdLD, JCCL, LH, CSH, AK, MM and FP working together as a group) repeatedly read the statements and quotations to identify and categorise needs. Together the group scrutinised the categories identified, and adapted, highlighted and grouped them into core categories linked to a conceptual framework of 'needs'. This data-led analysis was an iterative process: Theoretical insights from one part of the data were applied to other parts allowing new issues to emerge with re-reading and review, enabling the development of a framework to describe the diversity of needs expressed in the set of reviewed papers (rather than testing a previously developed hypothesis as in theory-led analysis)[3] Finally the reviewers re-read the statements of needs extracted from the studies to ensure that all were fully represented in the framework. PRISMA standards for reporting systematic reviews have been followed for this paper [4].

## Results

**Figure 1:**
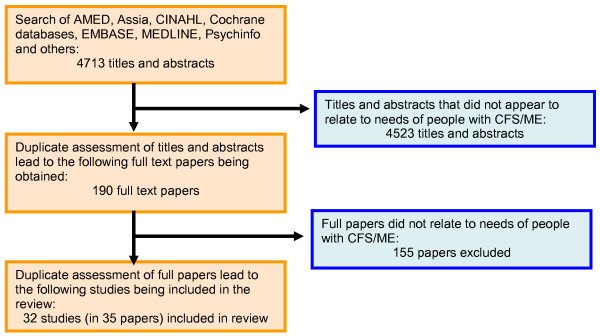
**Flow diagram of structured searches and inclusion assessment of studies**.

A total of 4713 titles and abstracts were reviewed; 190 full text papers were assessed in duplicate, and 32 studies (published in 35 papers) were included in this review. Figure 1 shows the flow of studies and Table 1 presents the characteristics of the included studies. The studies conveyed the needs of 2788 people with CFS/ME, plus many more in over 180,000 on-line discussion postings[5] All included studies were conducted in North West Europe, North and Central America, Australia and New Zealand. The majority of participants were women, but 8 studies included children and young people and many included men. Many studies did not report ethnicity or socio-economic status, but five included people from a range of educational[6,7] and ethnic backgrounds[8,9] or were conducted exclusively with minority ethnic groups [10-12] Nine studies did not report illness characteristics, but those which did indicated that the review covered a range of illness duration. Almost all of the 22 studies that specified illness severity included those with moderate or severe illness, two suggested a 'range' of severity. Four of the studies [13-16] were personal stories (or 'own stories' - not interpreted by a researcher but told directly as a story), see table 2.

**Table 1:** Characteristics of included studies

Study	Country	Number of people with CFS/ME*	CFS/ME illness		Age (years)	Female gender	Ethnicity and Socioeconomic status
						
			Duration (years)	Severity			
Anonymous 1997[13]	United States	1	NR	Moderate to severe	Adult	100%	NR

Asbring 2002[24]; Asbring 2004[21]	Sweden	12	1 to 23	A range: full time employed to sick leave, temporary disability or sickness pensions.	32 to 65	100%	NR

Ashby et al 2006[27]	United Kingdom	10	0.4 to 2	NR	8 to 16	70%	NR

Blake 1993[14]	Canada	1	NR	Severe	29	100%	Caucasian, well educated

Carlsen 2003[22]	Norway	5	NR	NR	23 to 67	80%	NR

Clarke 1999[6]; Clarke & James 2003[7]	Canada	59	A range	80% Severe; 20% moderate	18 to 80	65%	Ethnicity NR; a range of education levels

Denz-Penhey 1993[18]	New Zealand	10	NR	NR	6 to 18	NR	NR

Dumit, 2006[5]	United States	NR (180,000 on-line discussion postings)	NR	NR	NR	NR	NR

Edwards et al 2007[8]	United Kingdom	8	<1	Moderate to severe	18 +	100%	White British, Chinese and mixed; Socioeconomic NR

Garralda & Rangel 2004[10]	United Kingdom	28	NR	Moderate to severe, but all at school or home tuition	10 to 18	78%	A range of ethnicity and social class

Gray & Fossey 2003[35]	Australia	5	2 to 10	Severe	16 to 44	NR	NR

Green et al 1999[11]	United States	44	Mean 4.1	NR	18 to 57	89%	A range of ethnicity; socioeconomic NR

Hammond 2002[34]	United Kingdom	586	NR	NR	NR	NR	NR

Hoad 1994[15]	United Kingdom	1	3	Moderate	fifties	100%	White British; Socioeconomic NR

Horton-Salway 2004[28]	United Kingdom	15	NR	NR	NR	NR	NR

Jackson 1994[31]	United Kingdom	3	2 to 6	Severe	20 to 51	100%	NR

Jason et al 1996[40]	United States, Canada, Mexico	984	NR	A range	18 to 84	99%	NR

Lee et al 2001[30]	Canada	50	NR	Severe	20 to 64	56%	NR

Moore 2001[37]	United States	1	8	Moderate	NR	100%	Ethnicity NR; High education

Ong et al 2005[19]	United Kingdom	1	17	Severe	NR	100%	NR

Prins et al 2004[32]	The Netherlands	268	2 and more	NR	18 to 60	78.5%	NR

Rangel et al 2000[41]	United Kingdom	25	More than 3	Severe	12 to 20	NR	NR

Reynolds and Vivat 2006[26]	United Kingdom	3	16 and more	Moderate to severe	51 to 62	100%	White British; high education and 'home-maker'

Richards *et al *2006[33]	United Kingdom	21	More than 3	Moderate to severe	11 to 20	62%	NR

Roche & Tucker 2003[23]	United Kingdom	≈ 474	A range	NR	12 to 20	NR	NR

Schoofs et al 2004[29]	United States	46	NR	NR	18 and more	91%	NR

Schweitzer et al 1995[36]	Australia	47	NR	Moderate to severe	26 to 50	70%	NR

Sutton 1996[17]	United Kingdom	2	15 to 30	Moderate to severe	NR	50%	NR

Taylor & Kielhofner 2003[12]	United States	1	3	Moderate	28	100%	2^nd ^generation Irish immigrants; High education

Taylor 2004[9]	United States	47	NR	Moderate to severe	mean 49	96%	A range of ethnicity and social class

Weisstein, 2006[16]	United States	1	26	Extremely Severe	About 50	100%	Non- minority; High education

Whitehead 2006a[25]; 2006b[20]	United Kingdom	17	A range	Severe	13 to 63	65%	NR

NR: not reported

**Table 2:** Risk of bias of included studies

Study	**Study design, scope for expression of own needs in data collection**^1^	**Data analysis, scope for identification of needs**^2^
Anonymous 1997	**High**: Narrative of own story, detailed, personal and angry account	**High**: Reported without formal analysis

Asbring 2002, Asbring 2004	**Medium**: Case study; semi-structured interviews (60-150 minutes) to describe the Participants' encounters with their health care providers and possibilities of practicing the participants' power;	**High**: Thematic analysis using grounded theory, quotes presented

Ashby et al 2006	**Medium**: Case study; interviews and Likert-style rating scales	**Medium**: Content analysis; explicit interpretation by the authors, no quotes from participants

Blake 1993	**High**: Narrative of own story	**High**: Reported without formal analysis

Carlsen 2003	**Medium**: Case study; in depth interviews (open interview guide) plus observation of and participation in self help group meetings, plus data from health professionals and social workers,	**High**: Thematic analysis; quotes presented.

Clarke 1999, Clarke & James 2003	**Medium**: Case study; open-ended semi-structured telephone interview	**High**: Thematic analysis using constant comparative method, separately analysed for men and women; quotes presented.

Denz-Penhey 1993	**High**: Case study; participative action research with cycles of planning, action, observation and reflection with collaboration and participation of the participants; Interviews, statements, field notes, journal entries and questionnaires	**Medium**: ethnographic and action research; report of interpretation by the authors.

Dumit 2006	**High **This study drew on entries from internet newsgroup postings (180,000 internet entries), fieldwork and published debates; first person accounts, already in the public domain in internet newsgroups.	**High**. Thematic analysis conducted by the authors, early arguments submitted online and on scientific conferences and amended accordingly; quotes presented.

Edwards et al 2007	**Medium**: Case study; in-depth semi-structured interviews (1-1.5 hours)	**High**: Interpretative phenomenological analysis; quotes presented.

Garralda & Rangel 2004	**Medium**: Case study; semi-structured interviews with children with CFS and their parents (also children with arthritis and emotional disorders), and standard questionnaires. Note: This review used only the data on children with CFS/ME	**Low**: mostly statistical analysis of standardized questionnaire. (percentages and 3-group Kruskal-Wallis for categorical and Mann-Whitney for continuous data comparison).

Gray & Fossey 2003	**Medium**: Case study; semi-structured interviews (purposive sampling, videotaped interviews between participants and OTs, then individual interviews)	**High**: thematic analysis and quotes presented.

Green et al 1999	**Low**: Case study; postal standardised questionnaires on stigma, satisfaction in intimate relationships, labelling and symptoms' intensity.	**Low**: Statistical analysis (frequencies, percentages, correlations and Fisher's exact test)

Hammond 2002	**High**: Case study; in-depth interviews with claimants and non-claimants of DLA, combined with DLA data set and data from a survey with people with CFS/ME (posted questionnaires).	**High**: narrative analysis of in-depth interviews (quote reported); content analysis of DLA data set and posted questionnaires (percentages presented)

Hoad 1994	**High**: Detailed narrative of own story.	**High**: Reported without formal analysis

Horton-Salway 2004	**High**: Case study; naturalistic design, in-depth interview with a group member, and ethnographic observation of monthly meetings of a CFS support group and the talk to the group by a clinical psychologist.	**High**: Discursive analysis/thematic analysis; quotes reported.

Jackson 1994	**High**: Case study; in-depth interview	**High**: Discursive analysis; quotes reported.

Jason et al 1996	**Moderate**: Questionnaire sent out with the CFIDS Chronicle Journal containing open-ended questions on suggestions for improving services to people with CFS; standardized questionnaire on subjects' preferences on health services use (items developed from in-depth interviews).	**Low**: Descriptive statistics (frequencies, percentages, difference in means and standard deviations), factor analysis of the standardized questionnaire; quotes not reported.

Lee et al 2001	**High**: Case study; semi-structured interview, observational data and process notes over the course of the interviews, complementary quantitative data	**High**: Content and thematic analyses; quotes reported. Descriptive statistics of complementary data (percentages, means and standard deviations, medians),

Moore 2001	**Medium**: Single case study; data collection not reported; unclear whether data was collected via naturalistic observation or interview.	**High**: Narrative of the experience of a person with CFS/ME, reported by the researcher without formal analysis, with quotations.

Ong et al 2005	**High**: Case study; collaborative story-building of experience the development of GP-client relationship, based on own stories of a doctor and a client with CFS/ME.	**High**: Shared narrative of the experience by the two participants, commented by a researcher.

Prins et al 2004	**Low**: Posted questionnaires with closed questions and scales	**Low**: Means of social support compared between demographic and type of illness groups (CFS × others) and correlations. Changes in social support in a 14 month follow-up, MANOVA.

Rangel et al 2000	**Moderate**: Case study; case notes of 25 children with CFS/ME, followed by scales and semi-structured interviews with both children and parents.	**Low**: Description of mean and standard deviations; Mann-Whitney test, chi- square or Fish's exact tests to compare ill and recovered groups of children with CFS/ME

Reynolds & Vivat 2006	**High**: Case study; in-depth semi-structured interviews	**High**: Narratives analysis; quotes reported.

Richards et al 2006	**High**: Case study; tape recorded semi-structured interviews with 21 adolescents and their parents, carried out in the participants' houses.	**High**: content analysis with identification of themes; quotes reported.

Roche & Tucker 2003	**High**: Case study; questionnaires posted to young people with CFS/ME and their carers, one-to-one interviews with members of CFS/ME action group	**High**: Discourse analysis, quotes reported.

Schoofs et al 2004	**Medium**: Case study; semi-structured telephone interview and standard questionnaires of general health and health-related quality of life (SF36), quality of life questionnaire and perceived social support (PSSS).	**High**: Thematic analysis and descriptive categories using a comparative approach between participants with quotes reported. Bivariate correlations between scores of the questionnaires

Schweitzer et al 1995	**High**: Case-control study of person with CFS/ME compared with 30 undergraduate controls; semi-structured interviews and standardised questionnaire about sickness impact 'Sickness Impact Profile'	**Medium**: Thematic analysis of qualitative data, no quotes from participants. (Statistical results did not refer to expressed needs and were not extracted)

Sutton 1996	**High**: Case study; semi-structured interview with patients and GPs.	**High**: content analysis of interviews to identify themes and descriptive categories (quotes reported)

Taylor & Kielhofner 2003	**High**: Case study; in-depth interview and standardised questionnaires	**High**: Clinical interpretation of the case using patient's life history and information provided by scales of the domains of the MOHO. (quotes reported)

Taylor 2004	**Low**: Randomised controlled trial using standardised measures about quality of life and symptom severity	**Low**: repeated measures of ANOVA and regression analysis using random-effects to compare program and control conditions for two outcomes: quality of life and symptom severity.

Weisstein 2006	**High**: Narrative of own story with detailed personal accounts presented, data not analysed, quotations reported	**High**: Reported without formal analysis

Whitehead 2006a; Whitehead 2006b)	**High**: Case study; three one-to-one unstructured interviews over 2.5 years.	**High**: (2006a) Hermeneutic phenomenological analysis, quotes reported. (2006b) Narrative analysis to identify typologies of restitution, chaos and quest; quotes reported.

^1^*high*, if own story, participative action research or naturalistic observation; *medium*, if in-depth interview; *low*, if standardised questionnaire.

^2^*high*, if the paper presents participants' narratives or the study results present interpretations of narratives accompanied by quotes from participants; *medium *if interpretation was without quotes; and *low *if statistical analysis only.

Table 2 describes the risk of bias of included studies. Most studies provided high (n = 19/33) or medium (n = 11/33) scope for participants to express their needs. The data analysis and reporting of most studies provided high (n = 24/33) or medium (n = 2/33) scope for the reviewers to identify expressed needs. One study was designed primarily to examine expressed needs[17]

### Expressed needs of people with CFS/ME

**Figure 2:**
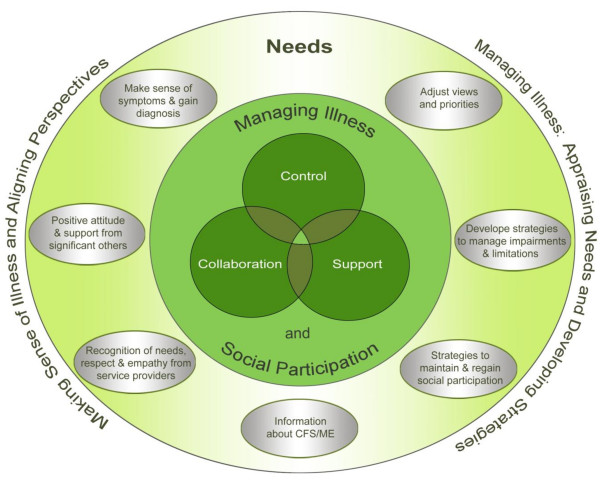
**Conceptual framework derived from the core categories of expressed needs of people with CFS/ME for support in managing the illness and social inclusion developed from the data within this systematic review**.

Figure 2 shows the conceptual framework of expressed needs of people with CFS/ME, developed from the 32 included studies. The framework indicates how expressed needs for managing the illness and social participation can be organised in two dimensions:

a) Making sense of the illness, gaining diagnosis and aligning perspectives

b) Managing the illness: appraising needs, developing strategies for needs to be addressed.

### The need to make sense of the illness, gain diagnosis and align perspectives

Making sense of the illness, gaining diagnosis and aligning perspectives were expressed as major needs throughout the course of the illness, but particularly during the early years when people with CFS/ME first encountered major life changes. In this context aligning perspectives refers to finding ways to adjust their own, and help significant others and health care professionals to adjust their, perspectives on life so that these may become similar or complementary.

This involved four key needs: i) making sense of symptoms and gaining diagnosis, ii) recognition of needs, respect and empathy from health and social care providers (iii) positive attitudes and support from significant others, and iv) information on CFS/ME.

#### i) The need to make sense of symptoms and gain diagnosis

Making sense of CFS/ME symptoms and gaining a diagnosis were crucial as many did not immediately recognise CFS/ME in themselves, and their symptoms were not understood by health professionals, family or friends[15,18] "*So many parts of my body were malfunctioning*" [19], but I had "*no idea what was wrong*", which was very frightening[14]

Not having a diagnosis posed challenges for relationships with friends, colleagues and relatives[20] People struggled to make decisions about which people could be given how much, and what type, of information about their health, selecting symptoms that appeared to have greater legitimacy. They worried that it would not seem credible to blame fatigue before a diagnosis was established [21] This sense of living with a mystery illness, lacking legitimacy, further compromised participation in many areas, such as family, work, leisure, health and social care.

Although diagnosis was perceived as crucial, the search for a diagnosis in adults and young people could take a long time - for some it involved more than ten years of consulting health professionals without being taken seriously[22,23]; or entailed long journeys - "*nearly 1000 miles to get to a doctor who could diagnose me*"[5] Some reported that doctors seemed not to believe in the existence of CFS/ME[14,24] To "*come to terms with the mystery illness and to get a diagnosis*"[14,15,25] "*was hard enough*"[15,20] and made them feel ill, frightened, angry and alone, bereft of support[14]

A diagnosis gave a name to their condition, opening a gateway for communicating needs, accessing support services and learning about their illness [9,14,17,18]. Once the diagnosis was confirmed, some experienced a tremendous sense of relief, even though at the time they did not know what the diagnosis meant[19] Although important, the diagnosis was also seen as bringing problems as "*learning that there is no explanation for a terrifying condition (known as CFS/ME) is devastating psychologically and socially*"[5]

#### ii) The need for recognition of needs, respect and empathy from service providers

Recognition of expressed needs by service providers was perceived as crucial to align perspectives and receive care needed to manage and gain control over their lives. People with CFS/ME emphasised the need for recognition that CFS/ME is a multi-faceted, disabling illness. Many reported that living with CFS/ME was particularly hard when doctors disbelieved in the illness[5], when CFS/ME was assumed to be a psychological illness, the breadth of symptoms and their impact were not fully acknowledged and/or were attributed to depression, psychosomatic conditions or malingering. In such situations they perceived themselves to be seen as 'hypochondriac', 'malingerers' or 'troublemaking'[5,16,26]

Disbelief and lack of empathy by health care providers emerged as common experiences and carried the threat of receiving only psychological treatment or not having their needs recognised as legitimate. This appeared to further compromise access to health and social care, and frequently led to a cycle of chaos. Many reported leaving a doctor's office in tears, knowing that they were very sick, but having no way to convince others about the legitimacy of their illness[5,11,14,19,22,24,27-31] Even in cases of severe pain or disability, people were told there was nothing wrong with them[16], "...*and if I couldn't walk, it was psychological*"[25] Others were told that recovery was a matter of getting up and pulling themselves together. "*I'll tell you what was said to me ....'Put your make up on, have your hair done and you'll feel a lot better ....I found it quite insulting"*[26]. People with CFS/ME felt that the battles they needed to fight for support made their lives more difficult: "*I think the plights of people with ME, the difficulties that are sort of added through the fights with benefit system and the health service"*[8]*"For the patient the process [of claiming long term disability benefits] is humiliating, exhausting and at times, results in a lengthier and more severe period of disablement"*[13].

The need to be treated as a whole person with a body, mind and spirit was another frequently unmet need. The lack of recognition of this need was especially serious for the severely ill, where their condition constrained their ability to communicate and take control of the management of their illness. People reported being "*treated like a non-person*"[5]. One woman, bed-bound for twenty five years with hypersensitivity to various stimuli, was not recognised as rational or aware: "'*Your wife's a vegetable' one helpful doctor shouted on the phone as Jesse [her husband] sat next to me six inches from my ear. It wasn't at all true...But on the outside, I could have been mistaken for a head of broccoli. I was almost completely locked in*"[16]

Lack of respect for, or belief in, people's self expression of health needs, was a major problem for those who were severely affected, disadvantaged or from minority groups. For example, a woman with terrible abdominal pain was not taken as a medical emergency with legitimate call on resource: "*Why was I having to act as my own medical spokesperson? What if I were too weak to talk? What if I were intimidated by the hospital because I was less well educated, or poor, or black in a sea of non-black higher authority, or English was my second language? Who would be my advocate then?*"[16] This lack of control affected health seeking behaviour. Weisstein[16] reported: "*I resisted going to the hospital, even though it was clear to me that something was horribly wrong. Every interaction and procedure in a hospital is fraught with terror: will this be the final push over the brink of 'post-exertional malaise'?"*

Although most reported experiences within the health and social services were negative, some people described examples of good practice. Health professionals conveyed messages of empathy, encouragement and personal commitment: by giving information and feedback, by demonstrating expertise about CFS/ME or a genuine interest in learning about it; by listening to, and discussing, available treatments, by encouraging the client to ask questions, not to suffer in silence and help others, like teachers, understand this illness and by informing where and how to find adequate care[19,25,27] Clinical psychologists and other health professionals were valued in providing psychological support to deal with this life-disrupting condition and the stigma associated with CFS/ME: "*the clinical psychologist who helped me begin to trust in myself again*"[14] Also important was demonstrating sympathy with the client's situation: *" [the doctor] just sat and looked at me with such compassion and empathy - I could have hugged him*"[19]

#### iii) The need for positive attitudes and support from family and friends

Support and understanding from family and friends was considered vital[14], and lack of social support was identified as a perpetuating factor of fatigue severity and functional impairment[32]. However, social isolation is often associated with CFS/ME[23] One young person commented "*The worst thing was not having any friends; it's important to have support from people who like you and give you confidence*"[27] The attitudes of significant others were crucial for young people: "*I think the single most helpful thing of all is when people don't judge*..."[33] and adults "*My husband has been a tower of strength and he understands, he's never questioned, he's never said ....you'll feel better soon. He understands and that has been very supportive*"[26]

Although health professionals were expected to facilitate positive attitudes of family and friends, some doctors caused others to dismiss symptoms leading to lack of support from family[19,30]:"*When my husband comes home from work, he always says, 'Why are you sleeping all the time? The doctor says there is nothing wrong*..."[30] The disbelief of significant others left them feeling bereft of support, frustrated and fearful[14,25]

#### iv) Needs for information relating to CFS/ME

Both before and after a CFS/ME diagnosis was established, information about the condition appeared central in allowing people to gain control of their lives. Some reported that medical knowledge acquired through friends' searches and emotional support helped them break a cycle of social withdrawal and disapproval,[5] whilst others offered doctors their own diagnosis[5,25] Discussions with, and feedback from, doctors during diagnosis helped people make sense of, and manage, their illness[14]

While diagnosis allowed access to formal support services, it was often difficult to find accessible services with up-to-date knowledge of CFS/ME and where professionals were sympathetic. Knowledge about CFS/ME, alongside support from significant others and empathy from health professionals, empowered people to get the care they needed. Health professionals conveyed useful information when listening and discussing available treatments and by providing information about where and how to find adequate care[19]

Equally important for many was information about, and help with, financial support. This need was vividly expressed, since for many "*their finances were severely strained*"[6] due to reduced job opportunities for themselves or their family carers and additional costs of support. Financial limitations in turn further limited social participation (as many social activities are expensive). While people with CFS/ME often felt forced to apply for disability benefits, they were not always able to demonstrate their eligibility[29,32,34]

ME support groups and associations were mentioned as valuable sources of information and contact with other people with CFS/ME[9,14,17,18] to help in grasping the "*strange, disabling but unpredictable nature of this condition*"[14,15] However, some had chosen not to take part in support groups as they could not find the energy to participate[18] or because "*identifying themselves and other people with CFS/ME primarily as people suffering from an illness" *would be detrimental to their positive thinking[24]

### The need to manage the illness, appraise their own needs and develop strategies to ensure that needs were addressed

Many people found that to have control over their lives and regain wellness with the confines of continued CFS/ME they had to adjust their views and priorities, to develop strategies to manage impairments and activity limitations and find ways to regain social participation[15,17,18,24]

#### v) The need to adjust views and priorities

Approaches to managing impairments ranged from trying almost anything in desperation, including pharmaceuticals, complementary medicine and diets,[25] while some younger people allowed CFS/ME to run its natural course[33] To come to terms with their illness and work towards wellness, many pointed out that they had to learn to recognize and address their whole self as a physical, emotional and spiritual being, as no one type of treatment could provide all the answers[14] "*No amount of knowledge can help someone to make the necessary internal adjustments; that has to do with attitude*"[15] Changes called for adjustments in self-valuation and attitudes, increasing awareness of limitations, decreasing focus on achievements[25] such as school grades[27], refocusing and re-prioritising relationships and health[25,31] This acceptance was often described as very difficult: "*It's been a very hard slog mentally to accept what I've got"*[8]

Accepting and learning to deal with limitations and changing lives could impose great challenges, which appeared to vary greatly between individuals and in differing periods of the condition. Disruption ranged from being able to maintain activities of daily living, employment, education and communication only at the cost of much effort and compromise in participative activities, to catastrophic limitations, as people found themselves in a wheelchair, housebound or bedbound. One man reported that "...*for someone who had been a self-sufficient, tax-paying, highly motivated individual all my life, having to now accept disability (which is also a humiliation, as I do not feel that I am earning it as I did when I was well)' was a great challenge*"[5] A first-hand account reports: "*I couldn't read, talk, listen, look, visit, or get up from a supine position. I had to wear a light-blocking mask over my eyes in a darkened room at all times. Nurses had to feed me. They had to whisper if and when they talked to me*."[16] Such limitations were closely related to restrictions on participation in relationships and personal expression. This was experienced as frightening, since well-being depended on carers to identify and respect their needs.

Accepting help and equipment, such as use of a dishwasher, a walking stick or employing a cleaner could therefore also pose a challenge[35] For others, befriending services[17], advocacy[16] and learning self-advocacy as part of independent living training proved helpful[9] As equipment and others' help could be seen as a badge of disability, they could pose difficulties for self-image and sense of independence despite being practically useful. "*I found it difficult to admit to myself that I needed help, and even more difficult to ask for it*"[15] The same woman described hiding a fold-up walking stick in a bag as "*such was my pride I tried desperately hard not to use it*".

Accepting psychological support appeared to help some people deal with the stressful experience of living with CFS/ME,[14,33] however, others saw accepting psychotherapy as implying that they had a psychological problem rather than a physical illness[33]

#### vi) The need to develop strategies to manage impairments and activity limitations

Many people with CFS/ME found themselves in a constant balancing act over how best to use their limited energy resources: "*the secret of coping is to accept that the imbalance exists, to weigh up the resources and to make a choice about how to use them*"[15] One study emphasised "*when energy is severely limited there is little time to spare for others and virtually none for outside the family*" [18]. This prioritisation may preclude access to forms of support such as CFS/ME self-help groups.

On occasions "*even sitting up in bed' could become too difficult*"[14] and a small increase in physical or mental activity could cause relapse "*with a vengeance*"[18] Rest and activity reduction could bring symptom relief[15,18,22,35,36], but was experienced as challenging and difficult to achieve[15] and often only brought temporary relief[18] Planned rest periods were important for many, including this young person: "*Sometimes I don't feel I need to rest, but I still rest because in the past I haven't felt I needed to rest, but then the next day I was really tired and it took longer for that to go"*'[33].

Finding meaningful occupation within the confines of the illness was important "*I certainly couldn't do anything like knitting. I couldn't concentrate on a pattern.... Eventually, I thought I'm fed up with just sitting watching television doing nothing, so I took up tapestry"*[26]. Adjustments were made by routinely appraising how successful their coping initiatives had been, in the context of self-knowledge and the perspectives of significant others. One person with CFS/ME reported having tried "*to do something every single day, like, otherwise [the illness] just walks all over me"*[35]

While activity-rest balance is important, it needs preparation and recovery time, making planning complex[15,22] Overdoing exercise was "*terrible, it makes me feel really ill."*, but gentle exercises to re-build muscles was helpful for some[33] For some young people, pacing activities, and gradually increasing activity, was useful when not linked to achievement pressure or over-riding good judgment about when to stop and rest[33] "*Don't do too much but set yourself targets. I didn't like being pushed and hated the idea of walking everyday, but it worked*"[27]

#### vii) The need to develop strategies to maintain/regain social participation

Impairments and activity limitations affected people's ability to maintain previous roles: "*you go through the grieving thing; it's the death of a whole lifestyle*"[29] Losses of multiple aspects of social participation seemed so painful that some people made huge effort to maintain their informal social life, work and educational roles[24,29] Leisure was an important need, often sacrificed to enable participation in employment and education, or as a direct consequence of impairments, economic disadvantage and social isolation. As relationships changed with friends, family and work, dealing with separation from former lives meant having to reassess and prioritise those relationships which were most supportive[7]

Outside the family, education was the major focus of social participation for many young people. Rarely attending school, some reported having lost connection with friends and teachers[23] Home tuition, a common alternative[10], allowed a flexible schedule of learning within the limits of their condition, but reduced social participation at an important stage of social development. Although intensive work required by school activities worsened the health of some children, others could ignore this effect, at least in the short term: "*I may think I am going to go into school so I may as well try and feel well*"[33] For other young people, school meant not having their needs acknowledged, being discriminated against and bullied by peers and educational workers who did not understand the complexity of their illness, considered them malingerers or lazy. School for children, and jobs for adults, were signifiers of living 'normally'[24], providing purposeful activity, an opportunity for social interaction, a sense of achievement, self-value and social recognition, income and social security[7,29]

However, needs stemming from work and education were substantial, posing difficult choices. Pacing work, by resting when fatigued, often meant taking work home to get it completed, and stressful decisions had to be made about disclosure; expectations of stigma made some conceal their illness[24] However, in a supportive environment, careful disclosure seemed helpful: "*They were very good and arranged a room with a bed in for me ....so I used to nap between lectures, that was the only way that I could get through the day*"[20]

In the absence of adequate social support, the effort of maintaining employment raised stress, compromised family roles and leisure activities or exacerbated the illness[36] Decisions therefore had to be made about who to disclose to, the extent of disclosure, and how far to limit activities[24] Others, severely affected by impairments, were discharged from their jobs when people learnt about their illness, or could not cope with working demands[12] - some "*broke down crying...as they described what they had had to give up.... For many that meant their jobs*"[29] Consequences included loss of earnings and radical lifestyle changes such as moving home as income reduced[15] Others lost access to recommended treatments as they could no longer pay[37]

## Discussion and Conclusion

Exploring the commonalities and relationship between the themes raised in the reviewed papers has highlighted several interrelated aspects of the needs of people with CFS/ME in regaining well-being and control over their lives - making sense of the illness, aligning perspectives, managing the illness, appraising needs and developing strategies for social inclusion and control. This review has also identified that many psychological and physical demands can be made on people with CFS/ME and that major needs may be largely unmet due to poor recognition of CFS/ME as an illness or of its impact. The review has shown that the lack of recognition of needs and poor support from the health and social systems further compromise socioeconomic status, activities of daily living, social participation and personal development, thereby exacerbating the impact of the illness in their lives.

### Strengths and limitations of the review

The primary studies examined in this systematic review were often not primarily designed to assess and discuss the needs of people with CFS/ME. This may mean that some important needs may have been missed in the reporting. However, whilst the included papers were often not primarily designed to examine needs, and used diverse methodologies, most provided some scope for participants to express their own issues. The conceptual framework of needs developed by data-led analysis enabled the diversity of needs expressed in the set of reviewed papers to emerge, and also facilitated the synthesis of studies that drew on a range of theories and approaches that were not all directly comparable. As well as providing insights on the support needs of people with CFS/ME, the review provides a basis for further research. While some studies explicitly reported the inclusion of disadvantaged socio-economic groups and ethnic minorities, none of the studies examined their specific experiences and needs. Most of the studies that discussed severity of the illness of included participants suggested that they had moderate and/or severe illness, so that the results of this review may not be applicable to those with mild illness.

The backgrounds of researchers inevitably colour their view of the material they work with, and this is true of systematic reviewers as well as primary researchers. Systematic review methodology was used to reduce some of the biases that reviewers pre-conceptions can create (such as partial inclusion of material depending on how well it resonates with pre-conceived ideas of the results or uncritical inclusion of only partially valid study conclusions). The diverse backgrounds of the reviewers also helped to prevent a single pre-conceived view to dominate (for example, MdLD focuses on social inequalities, JCdCL is a psychologist, LH is a systematic review methodologist and dietitian, CSH is an occupational therapist, DP is an epidemiologist, LN is a consultant in public health medicine and epidemiologist, EL specialises in occupational and public health and has interest in qualitative participatory methodologies, PC is a retired General Practitioner with an interest in CFS/ME, AK is an OT and researcher specialising in participative approaches, MM is an occupational therapist and qualitative researcher with specific interest in chronic health conditions, FP is a sociologist with an interest in community-based research and social support).

Despite the limitations of the reviewed articles, they proved a valuable source of evidence of needs felt and expressed by people with CFS/ME. This can be used in future service specifications and evidence based guidelines for management of CFS/ME. The review suggests that whilst CFS/ME presents particular challenges for formal needs assessment, the needs of people with CFS/ME can be identified, articulated and contextualised so that their relevance can be understood and appropriate care provided.

The review has not examined the validity of the expressed needs of people with CFS. One theory links the early parental environment with neurobiological development via the hypothalamo-pituitary adrenal axis, changing stress responsiveness through life in those with CFS/ME [38]. This could potentially result in increased 'neediness' in those with CFS/ME but would not invalidate those needs. As reviewers we have taken the needs expressed by people with CFS/ME at face value - even if their need for support is higher than in others, the needs of people with CFS/ME are expressed very consistently and their accounts of their needs deserve to be heard and responded to.

The review has shown that there are strongly conveyed and widely-shared needs for financial and functional support, for ameliorating fatigue, pain and other impairments from CFS/ME, and for support in refocusing lives in an environment of ignorance and social prejudice. The importance of financial support, and the difficulties in obtaining that support in the UK, have been recently highlighted in the Action for ME Welfare Reform consultation survey carried out in October 2008. This survey found that the health of 79% of 611 respondents were adversely affected by the process of benefit assessment, and 48% of 574 respondents stated that they had had a relapse as a result of this process[39] That so many of these needs are unmet is surprising in developed nations and may be considered unacceptable by many. The review has also shown that being listened to, believed, understood, respected and encouraged in a non-judgmental manner by people who know about their illness underpins almost all needs for support.

Gaining support appears to depend less on the ability of the person with CFS/ME to articulate their needs, but more on the ability of others to understand and respond to the holistic nature and specific needs of each individual. Addressing and managing such needs is essential to both the collaborative process of re-building lives as well as in limiting the burden of the illness on carers and local health and social services.

These needs are specific and substantial, but can be addressed by the body of good practice built in collaborations between people with CFS/ME and those supporting them, whether family, friends, patients' organisations or health and social care services. Guidelines for good practice should be backed up by public and professional education about CFS/ME, public investment in service provision and inter-sectoral policies (including health, education, work and pension, and housing) with an explicit agenda for acknowledging the illness known as CFS/ME, fulfilment of human rights, equitable care and social inclusion of people with disabilities.

## Competing interests

The authors declare that they have no competing interests.

## Authors' contributions

MdLD conceived the review and it was designed by MdLD, LH and FP. LH carried out the searches, and MdLD. LH, CSH, AK, MM and FP assessed the lists of titles and abstracts, collected the full text papers, assessed full text papers for inclusion and data extracted included studies. CSH, LH, MdLD, JCCL and FP assessed validity of included studies. MdLD, JCCL, LH, CSH, AK, MM and FP took part in group analysis and interpretation of the data. MdLD, LH, AK, MM and FP wrote parts of the first draft of the manuscript, all authors commented on and edited the manuscript, and all authors ageed the final version of the paper. LH submitted the review for publication.

## Appendix A

Medline search strategy (adapted for other databases)

1 chronic fatigue.mp.

2 fatigue syndrome$.mp.

3 cfs.mp.

4 myalgic encephalo$.mp.

5 PVFS.mp.

6 CFS$.mp.

7 cfids.mp.

8 exp fatigue syndrome chronic/

9 (unexplained adj3 fatigue).mp.

10 1 or 2 or 3 or 4 or 6 or 7 or 8 or 9

11 (health or education or school or college or university or work or legislation or mobil$).mp.

12 (activit$ adj5 living).mp.

13 (need or needs or impact or impacts).mp.

14 exp legislation/

15 mobility.mp.

16 exp rehabilitation/

17 exp education/

18 exp 'health services needs and demand'/

19 exp social behavior/

20 exp Disability Evaluation/

21 dysfunct$.mp.

22 11 or 12 or 13 or 14 or 15 or 16 or 17 or 18 or 19 or 20 or 21

23 10 and 22

## Pre-publication history

The pre-publication history for this paper can be accessed here:

http://www.biomedcentral.com/1471-2458/9/458/prepub
